# Author response to: Comment on: Cachexia index for prognostication in surgical patients with locally advanced oesophageal or gastric cancer: multicentre cohort study

**DOI:** 10.1093/bjs/znae173

**Published:** 2024-07-16

**Authors:** Leo R Brown, Andrew B Crumley, Richard J E Skipworth

**Affiliations:** Clinical Surgery, University of Edinburgh, Royal Infirmary of Edinburgh, Edinburgh, UK; Department of General Surgery, Forth Valley Royal Hospital, Larbert, UK; Department of General Surgery, Forth Valley Royal Hospital, Larbert, UK; Academic Unit of Surgery, University of Glasgow, Glasgow Royal Infirmary, Glasgow, UK; Clinical Surgery, University of Edinburgh, Royal Infirmary of Edinburgh, Edinburgh, UK

*Dear Editor*

We thank Arkle *et al*.^1^ for their interest in our study^2^ and are happy to discuss the points they raised.

Body composition analyses conducted using ‘Data Analysis Facilitation Suite’ (DAFS) provide high levels of accuracy and have previously been validated against manual segmentation. DAFS provides a less labour-intensive alternative to the other software types (for example ImageJ, developed at the National Institutes of Health) most widely used within the literature. From our experience of analysing several thousand CT images, across this and other studies, we can reassure Arkle *et al*.^1^ that we have observed low rates of segmentation errors requiring manual edits by the reviewing clinician. Such issues tend to occur when an artefact disrupts the image (for example surgical drains), which is unlikely in this preoperative setting. Use of body composition analysis is almost exclusively restricted to the research arena at present and future integration of this technology into existing platforms (for example Carestream Picture Archiving and Communication System) would likely be beneficial for ensuring consistency. We hope that findings from our work, and similar studies, might help justify such a development.

**Fig. 1:**
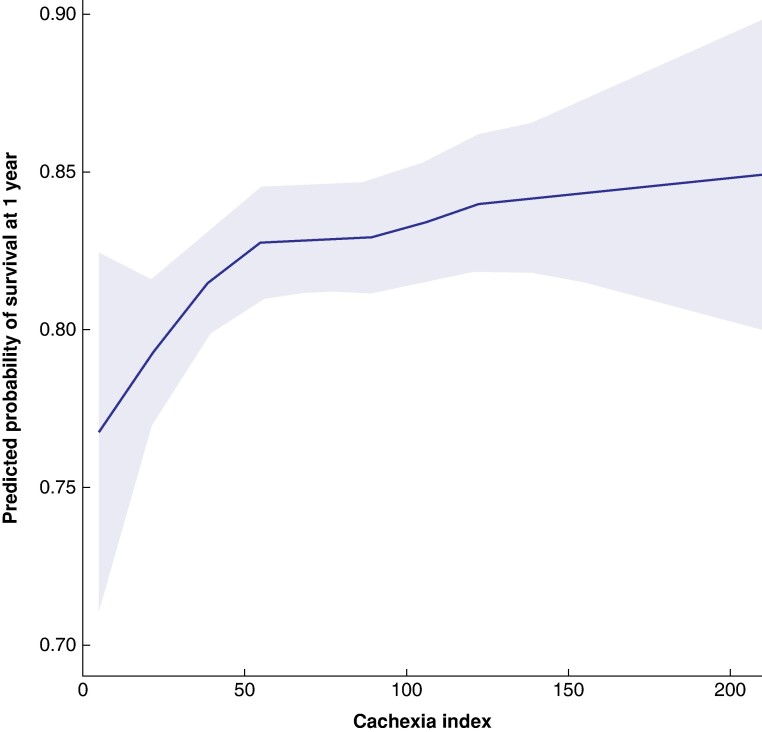
Survival probability across a range of cachexia index values

We opted for a single ‘cut-off’ after careful consideration of the data. Having explored incremental categories (tertiles, quartiles, and quintiles), it was consistently noted that the adverse effect of the cachexia index (CXI) was only apparent in those patients with the lowest values. When survival probability is plotted across a continuous range of CXI values (*Fig. 1*), we can see that it is not a straightforward linear relationship. As such, handling CXI as a continuous variable (drawing one regression line through it) would also not accurately convey that relationship.

We absolutely agree with the comments of Arkle *et al*.^1^ regarding the potential for a low CXI to be associated with disease that has been clinically under-staged. Additional investigation amongst this group may indeed make a worthwhile study idea. We would also agree that cut-offs stratified by tumour site could further improve the marker’s utility and would encourage exploration of this across larger-scale studies.
